# Targeting early B-cell receptor signaling induces apoptosis in leukemic mantle cell lymphoma

**DOI:** 10.1186/2162-3619-2-4

**Published:** 2013-02-19

**Authors:** Mohand-Akli Boukhiar, Claudine Roger, Julie Tran, Remy Gressin, Antoine Martin, Florence Ajchenbaum-Cymbalista, Nadine Varin-Blank, Dominique Ledoux, Fanny Baran-Marszak

**Affiliations:** 1 INSERM, UMR U978, Adaptateur de Signalisation en Hématologie, F-93000, Bobigny, France; 2 Université Paris 13, Sorbonne Paris Cité, Labex INFLAMEX, F-93000, Bobigny, France; 3 AP-HP, Service d’hématologie biologique, Hôpital Avicenne, F-93000, Bobigny, France; 4 AP-HP, Service d’Anatomopathologie, Hôpital Avicenne, F-93000, Bobigny, France; 5 INSERM, U823, Université Joseph Fourier, Institut Albert Bonniot, F-38000, Grenoble, France; 6 CHU de Grenoble, Service d’hématologie clinique, F-38000, Grenoble, France; 7 French GOELAMS Group, UMR U978 INSERM-Université Paris 13, UFR SMBH, 74 rue Marcel Cachin, 93000, Bobigny, France

**Keywords:** Mantle cell lymphoma, LYN, BCR, EGR-1, Dasatinib

## Abstract

**Background:**

We previously showed that B-cell receptor (BCR) signaling pathways are important for *in vitro* survival of mantle cell lymphoma (MCL) cells. To further identify early BCR-activated signaling pathways involved in MCL cell survival, we focused our study on BCR-proximal kinases such as LYN whose dysregulations could contribute to the aggressive course of MCL.

**Methods:**

Primary MCL cells were isolated from 14 leukemic patients. Early BCR-induced genes were identified by qRT-PCR array. The basal and BCR-induced phosphorylation of LYN and JNK were evaluated by immunoblottting. Cell survival signals were evaluated by apoptosis using flow cytometry.

**Results:**

We showed that LYN was constitutively phosphorylated in MCL cell lines and in 9/10 leukemic MCL cases. Treatment with dasatinib or with a specific inhibitor of Src kinases such as PP2 suppressed constitutive LYN activation and increased *in vitro* spontaneous apoptosis of primary MCL cells. BCR engagement resulted in an increase of LYN phosphorylation leading to activation of c-JUN NH2-terminal kinase (JNK) and over-expression of the early growth response gene-1 (EGR-1). Inhibition of JNK with SP600125 induced apoptosis and reduced level of basal and BCR-induced expression of EGR-1. Furthermore, decreasing EGR1 expression by siRNA reduced BCR-induced cell survival. Treatment with PP2 or with dasatinib suppressed BCR-induced LYN and JNK phosphorylation as well as EGR-1 upregulation and is associated with a decrease of cell survival in all cases analysed.

**Conclusions:**

This study highlights the importance of BCR signaling in MCL cell survival and points out to the efficiency of kinase inhibitors in suppressing proximal BCR signaling events and in inducing apoptosis.

## Background

Mantle cell lymphoma (MCL) constitutes about 6%–10% of non-Hodgkin lymphoma and despite recent advances in the treatment, the disease has not generally been cured with a poor progression-free survival for a large number of patients. New therapies that target specific signaling molecules are therefore of potential value. In recent years, some studies tried to reveal new suitable therapeutic targets [1-9] and have clarified the impact of several signaling pathways for increased proliferation and resistance to apoptosis of MCL cells. Constitutively active B-cell receptor (BCR)-mediated signaling has been implicated in the pathogenesis of a number of NHLs including diffuse large B cell lymphoma (DLBCL), follicular lymphoma, gastric mucosa-associated lymphoid tissue lymphoma and B-cell chronic lymphocytic leukaemia (CLL) [10-13]. Recently, we demonstrated in primary MCL cells a central role for active BCR signals in survival of MCL cells [14]. The activated forms of the BCR-associated kinases LYN and spleen tyrosine kinase (SYK) were present in MCL tumor tissues therefore supporting an *in vivo* role of active BCR signaling in this pathology [15]. Moreover, MCL is characterized by a highly restricted immunoglobulin gene repertoire with stereotyped VH CDR3s and precise Somatic Hyper Mutation targeting, thus strongly implying a role for antigen-driven selection of the clonogenic progenitors [16].

Upon antigen engagement, Igα–Igβ heterodimer (CD79a-CD79b) are phosphorylated on immunoreceptor tyrosine-based activation motif (ITAM) tyrosines by the BCR-associated kinase LYN, which belongs to the Src family kinases (SFK). SYK protein is then recruited through its SH2 domain to the phosphorylated Igα–Igβ heterodimer, resulting in the triggering of different signaling cascades [17]. Among them, the PLCγ2/PKC pathway is crucial for activation of various mitogen-activated protein kinases (MAPKs), such as extracellular signal-regulated kinase (ERK) and c-JUN NH2-terminal kinase (JNK). Extensive work by several groups has established that MAP kinase pathways play critical roles in the pathogenesis of various hematologic malignancies, providing new potential molecular targets for future therapeutic approaches [18]. Indeed, gene expression profiling of DLBCL revealed enhanced expression of JNK mRNA in at least 60 percent of cases [19]. Moreover inhibition of JNK activation by the pharmacological inhibitor SP600125 induced growth arrest in myeloma cell lines [20]. Of interest, JNK was showed to be constitutively activated in MCL and inhibition of phospho-JNK with SP600125 resulted in growth arrest in MCL cell lines (Jeko-1, HBL-2, UPN-1, Granta-519) [21].

A key downstream target of JNK activation is the early growth response gene-1 (EGR-1) transcription factor playing an important role in cell cycle regulation, cell proliferation and apoptosis [22,23]. EGR-1 was first identified as a putative G0/G1 switch regulatory gene in lymphocyte cultures [24]. Constitutive EGR-1 expression is involved in the self-renewal capacity of B-1 lymphocytes [25] and hematopoietic stem cells [26]. EGR-1 is also constitutively expressed in immature BKS-2 B lymphoma and inhibition of EGR-1 using specific antisense oligonucleotides induced apoptosis [22]. Alternatively, mature B2 cells undergo proliferation with an increase of EGR-1 expression upon BCR engagement [25]. Moreover, EGR-1 is down-regulated upon JNK inhibition by SP600125, and its overexpression partially protects against JNK inhibitor-induced apoptosis in B lymphoma cell lines [27].

Given the importance of BCR signaling in tumor cell survival including MCL cells, we hypothesized that targeting BCR-associated kinases such as SFK represents a potentially useful strategy to treat MCL. LYN kinase is the major SFK expressed in B cells and its constitutive phosphorylation was previously reported in Jeko-1 cell line [28]. However its role in MCL has not yet been explored to date. Therefore we analysed the activation status of LYN in primary MCL cells and evaluated the *in vitro* impact of its inhibition on MCL cells survival. We showed that LYN was constitutively phosphorylated in most MCL cases tested and that BCR engagement led to an increased LYN phosphorylation. Treatment with dasatinib, the oral broad inhibitor of tyrosine kinases, suppressed BCR-induced LYN and JNK phosphorylation in primary MCL cells. Similarly, treatment with dasatinib inhibited BCR-dependent EGR-1 upregulation and cell survival. Using PP2, a more specific inhibitor of BCR-associated SFK, we proved the efficiency of blocking BCR-emanating signals in suppressing MCL cell survival.

## Results

### EGR-1 and c-MYC are rapidly induced upon BCR engagement in MCL

We have previously described that BCR engagement induces a survival signal in MCL through an IL6/IL10-dependent activation-loop of STAT3 [14]. To further investigate which BCR-induced signaling pathways are critical, we screened purified B cells from primary leukemic MCL for the differential expression of 84 genes upon anti-IgM stimulation using RT^2^ Profiler PCR Arrays (SA Biosciences PAHS-027). Fifteen genes exhibited significant increased or decreased expression as compared to unstimulated cells (Additional file 1: Table S2; average fold-change from 4 individual experiments using UPN 5, 10, 13 and 14). Four genes were down-regulated (APAF1, CCNG2, ATM, BTG2), all corresponding to proapoptotic proteins. Conversely, eleven genes were overexpressed (CDK4, MCL1, NFkB1, IFNB1, SESN2, BCL2A, IL6, TNF, HK2, EGR-1, c-MYC), all of them being involved in cell cycle progression or inhibition of apoptosis. Within this group, three genes encoded for transcription factors; namely NF-kB, c-MYC and EGR-1 the two later being the two most upregulated genes upon anti-IgM stimulation (average of 23.6- and 17.6-fold increase, respectively).

**Figure 1:**
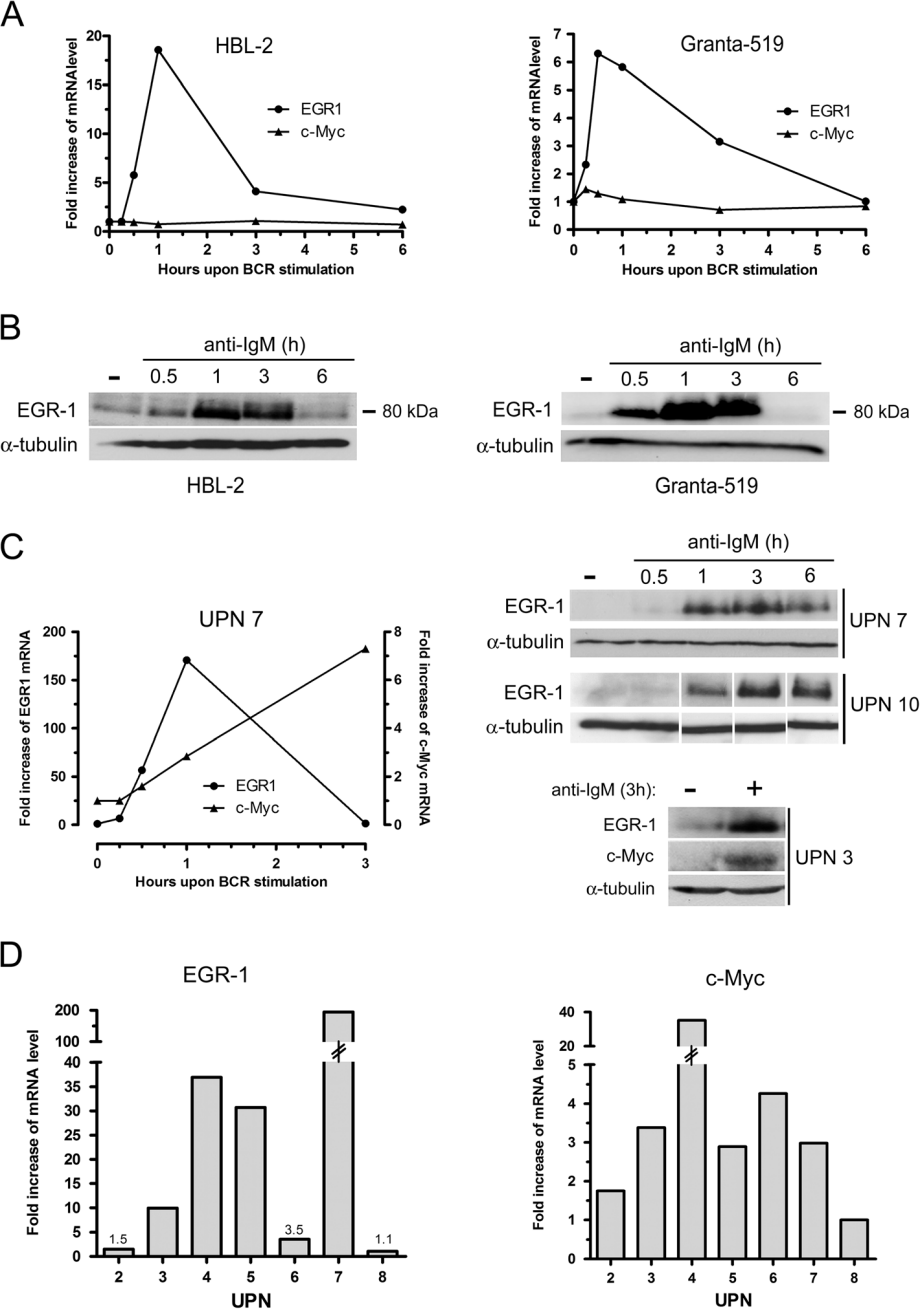
**Kinetics of BCR-induced expressions of EGR-1 and c-MYC in MCL cell lines and primary MCL cells.** (**A**) Kinetics of BCR-induced mRNA expression of EGR-1 and c-MYC in Granta-519 and HBL-2 cell lines. 3×10^6^ cells/ml were stimulated with 10 μg/ml of immobilized anti-IgM antibody for 15 min, 30 min, 1 h, 3 h and 6 h. EGR-1 and c-MYC expressions were analyzed by qRT-PCR. (**B**) Induction of EGR-1 protein upon BCR engagement was confirmed by western-blot. (**C**) The same experiments were performed on primary patients’ cells. EGR-1 and c-MYC expressions were analyzed by qRT-PCR (left panel) and western-blot (right panels). (**D**) EGR-1 and c-MYC mRNA expressions upon anti-IgM stimulation (1 h) were analyzed by qRT-PCR from 7 patients’ samples (UPN2, 3, 4, 5, 6, 7 and 8). Fold increase of mRNA level were calculated compared with unstimulated cells in all experiments. All measurements were done in duplicate and the mean is provided.

BCR-induced expressions of c-MYC and EGR-1 were then confirmed by kinetic experiments in MCL cell lines (Granta-519 and HBL-2) (Figure 1A and B) and in MCL patients’ samples (Figure 1C). For MCL cell lines, basal levels of EGR-1 mRNA was rapidly increased within 30 min upon BCR ligation, peaked at 1 h and gradually returned to basal level within 3 to 6 hours. Similarly, EGR-1 protein levels increased upon anti-IgM stimulation and returned to basal level within 6 h (Figure 1B). A similar increase was observed for primary cells (UPN7 and UPN10) with EGR-1 proteins still detectable at 6 hours (Figure 1C). C-MYC expression was significantly induced upon BCR engagement in patients’ cells only (Figure 1A for Granta-519 and HBL-2 cells, Figure 1C for UPN7). The pattern of c-MYC mRNA induction differed from that of EGR-1 and displayed a constant increase at least up to 3 h associated with an increase of c-MYC protein (Figure 1C). We next evaluated the impact of BCR stimulation on a series of 7 patients’ samples (Figure 1D). Upon 1 h of anti-IgM stimulation, EGR-1 mRNA expression was highly upregulated in 4 out of 7 cases and clearly with a major extent as compared to induction of c-MYC (median fold increase: 30.1 and 2.98 for EGR-1 and c-MYC respectively). No correlation was observed between IGHV mutational status and intensity of BCR-induced responses (not shown).

### Inhibition of JNK suppresses both BCR-induced EGR-1 upregulation and cell survival

**Figure 2:**
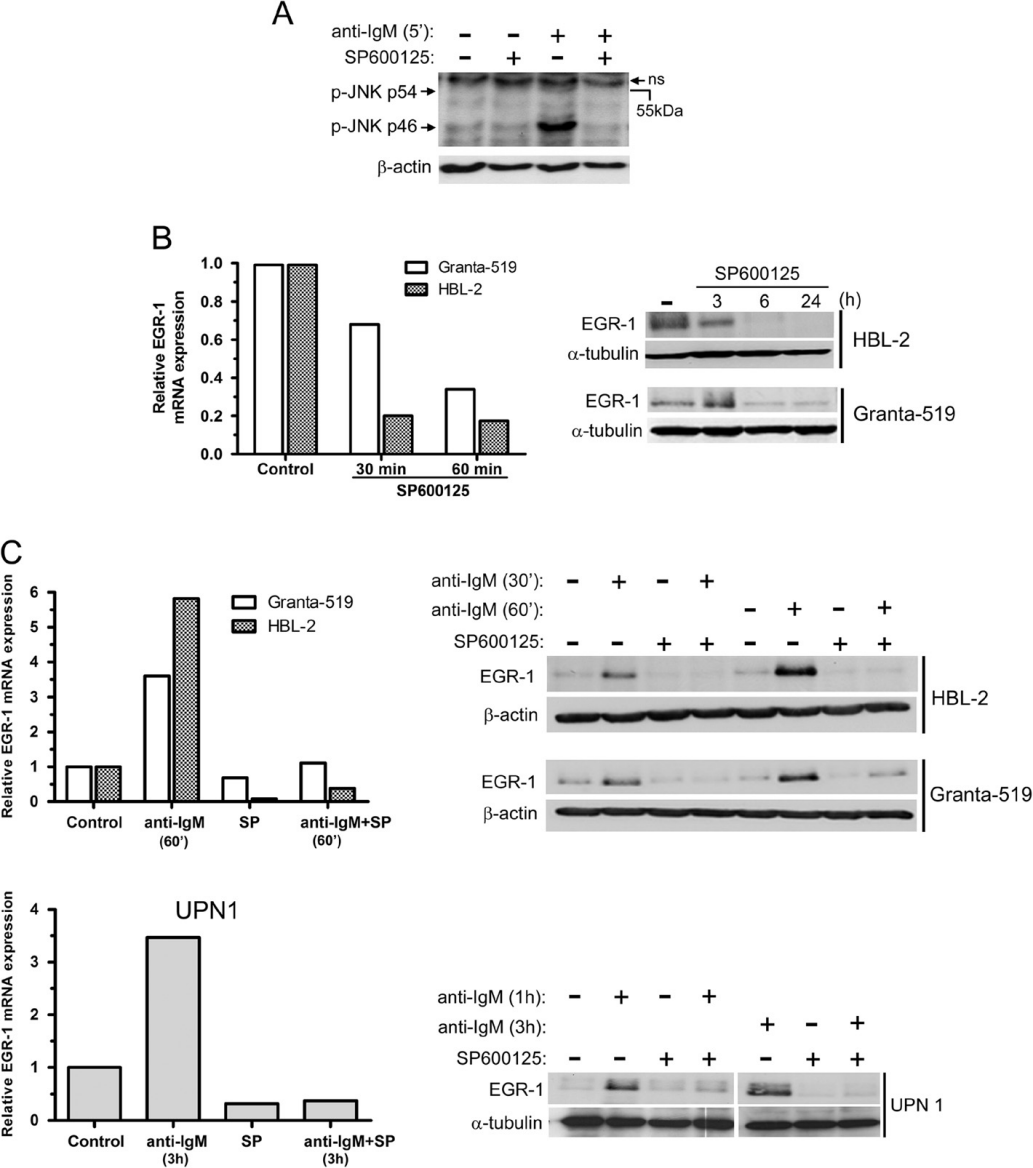
**Basal and BCR-induced EGR-1 expressions are dependent on JNK activation.** (**A**) Primary cells (UPN9) were pretreated with SP600125 (10 μM) for 1 hour and then stimulated with soluble anti-IgM antibody (10 μg/ml) for 5 min. Basal and BCR-induced phosphorylation of JNK (p54 and p46) were analyzed by western-blot. ns: non specific band. (**B**) Treatment with SP600125 led to a time-dependent decrease of mRNA and protein EGR-1 levels in Granta-519 and HBL-2 cells. (**C**) Impact of SP600125 on BCR-induced EGR-1 expression. Granta-519, HBL-2 and primary cells (UPN1) were pretreated with SP600125 for 1 h and then stimulated with immobilized anti-IgM antibody. EGR-1 mRNA and protein levels were analyzed by qRT-PCR and western-blot respectively. Fold increase of mRNA level were calculated relative to unstimulated cells in all experiments. All measurements were done in duplicate and the mean is provided.

Since EGR-1 has been described as a downstream target of JNK activation in various cellular models, we analyzed in MCL the involvement of JNK in the BCR-induced upregulation of EGR-1 and its role on MCL cell survival. In a characteristic patient sample (UPN9), basal JNK phosphorylation was slightly detected and was further enhanced following 5 min of BCR ligation with higher increase of phospho-JNK p46 (Figure 2A). Moreover, increase of BCR-induced phospho-JNK p46 was fully abolished in the presence of a selective inhibitor of JNK (SP600125, 20 μM) (Figure 2A). Inhibition of JNK by SP600125 induced a rapid down-regulation of EGR-1 mRNA expression in HBL-2 and Granta-519 cells associated with a subsequent decrease of EGR-1 protein (Figure 2B). Moreover, treatment with SP600125 upon anti-IgM stimulation also led to a blockade of BCR-induced EGR-1 upregulation in MCL cell lines (HBL-2 and Granta-519) and in primary MCL cells (UPN1) (Figure 2C). To confirm that EGR-1 was a downstream target of JNK in response to BCR activation, anti-IgM-stimulated HBL-2 cells were incubated with 5Z-7-Oxozeanol, an inhibitor of the transforming growth factor-β activated kinase-1 (TAK1) that is critical for BCR-induced JNK activation in B cells [29]. As shown in Additional file 2: Figure S1, treatment with 5Z-7-Oxozeanol (0.5 μM) completely abrogated BCR-induced upregulation of EGR-1. Overall, these results indicate that constitutive and BCR-induced EGR-1 expressions are dependent on JNK activation in MCL cells.

**Figure 3:**
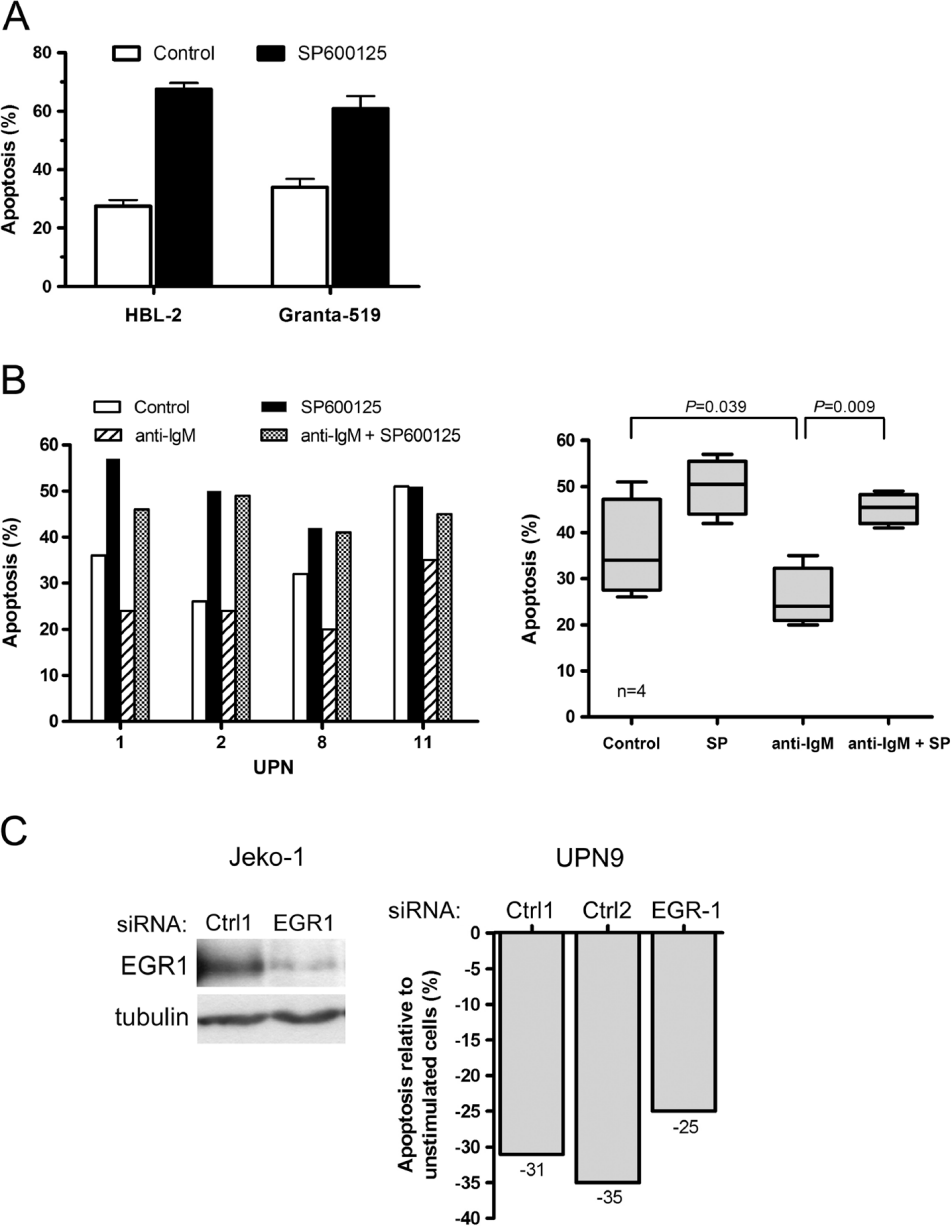
**Targeting JNK and EGR-1 induces MCL apoptosis and decreases BCR-induced cell survival.** (**A**) HBL-2 and Granta-519 cells were treated with SP600125 for 48 h and apoptosis was measured by flow cytometry. Percentage of apoptotic cells corresponded to% of annexin V-positive, including PI-negative and PI-positive cells. Mean ± SD of 2 independent experiments is represented. (**B**) Patients’ cells (UPN1, 2, 8 and 11) were stimulated with immobilized anti-IgM for 24 h with or without SP600125 (10 μM) and the percentage of apoptotic cells was determined by flow cytometry after gating on CD19+ cells (*left panel*). All measurements were done in duplicate and the mean is provided. Results are also shown as median ± quartile (*box*) ± SE (*bars*) (n=4) (*right panel*). Differences between groups were determined using the paired Student *t* test. (**C**) Jeko-1 cells were transfected either with control siRNA (Ctrl1) or EGR-1 siRNA and EGR1 protein level was determined by western-blot after 72 h of culture (*left panel*). Primary MCL cells (UPN9) were transfected either with controls siRNA (Ctrl1, Ctrl2) or EGR-1 siRNA and subsequently stimulated with anti-IgM for 24 h or left unstimulated (*right panel*). Percentage of apoptotic cells was normalized to unstimulated cells and calculated as follows: [(% apoptosis BCR stimulated cells -% apoptosis BCR-unstimulated cells) / (% apoptosis BCR-unstimulated cells)] x100. All measurements were done in duplicate and the mean value is provided.

We next investigated the impact of JNK inhibition on MCL cell survival. Treatment of HBL-2 and Granta-519 cells with SP600125 for 48 h increased apoptosis (from 27% and 34% to 68% and 61% of apoptotic cells for HBL-2 and Granta-519, respectively) (Figure 3A). A similar increase of apoptosis was observed in MCL primary cells (36 ± 5% and 50 ± 3% for untreated and treated cells respectively, n=4) (Figure 3B). Moreover, BCR engagement induced in most cases a significant inhibition of spontaneous apoptosis (p=0.039) that was abrogated by a treatment with SP600125 (p=0.009) (Figure 3B). To confirm the involvement of EGR-1 in BCR-induced cell survival, MCL primary cells transfected with EGR-1 siRNA were stimulated with anti-IgM. As shown in Figure 3C, a reduction of 20% to 30% of cell survival was observed as compared to transfection with control siRNA. Collectively, these results indicate that EGR-1 is a downstream target of JNK in MCL cells and that JNK promoted constitutive and BCR-induced cell survival in MCL implicating notably EGR-1 induction.

### Inhibition of LYN activity is associated with an increase of apoptosis in MCL cells

**Figure 4:**
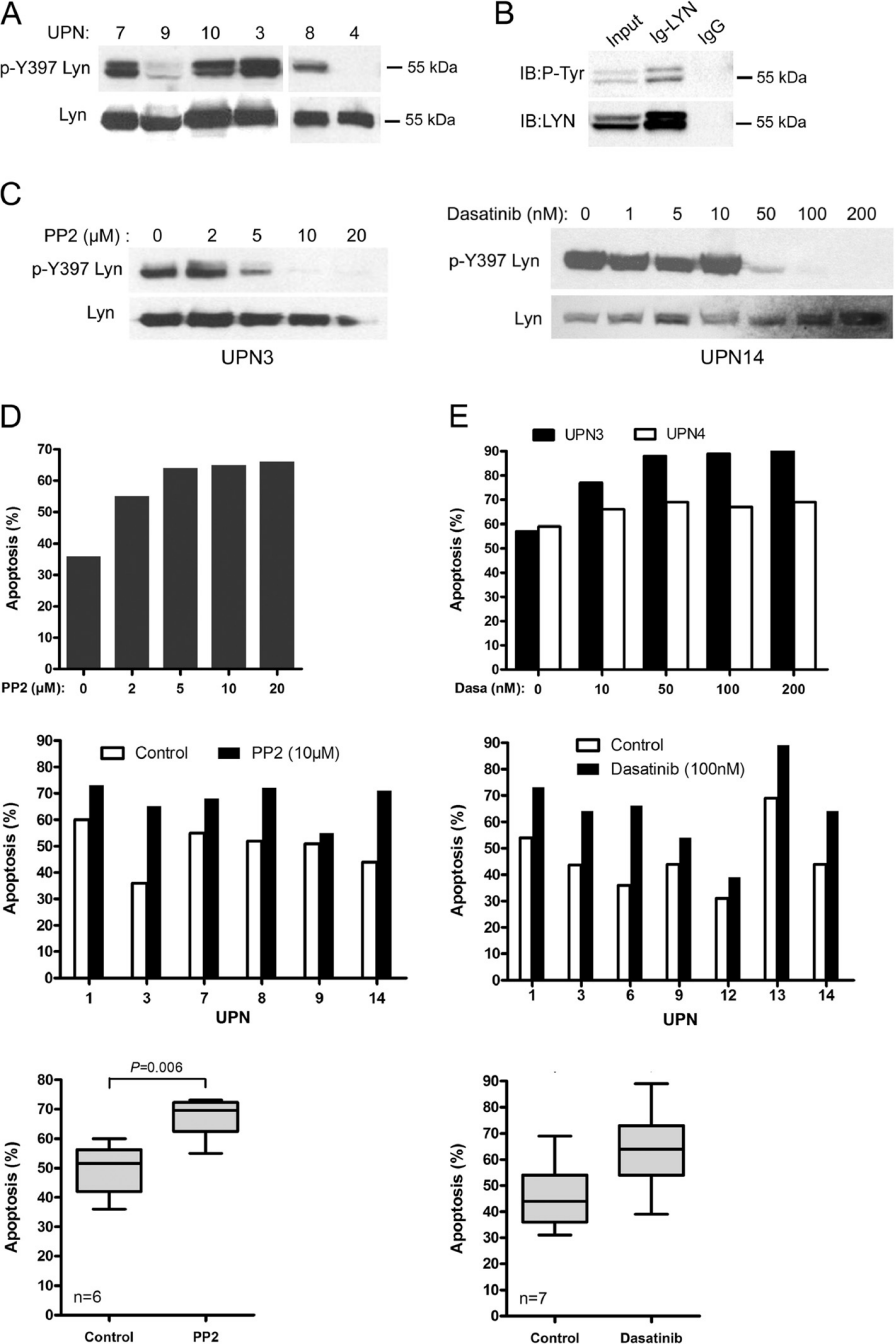
**PP2 and dasatinib inhibit constitutive phosphorylation of LYN and induce apoptosis of primary MCL cells.** (**A**) Constitutive phosphorylation profiles of LYN in MCL patients’ samples. Phospho-Tyr397 LYN was detected using a pan phospho-src family antibody. The blots were stripped and re-probed for total LYN. (**B**) Total proteins from HBL-2 cells were immunoprecipitated with an anti LYN antibody (Ig-LYN) or with an irrelevant IgG control and immunobloted (IB) with either an anti-phosphotyrosine antibody (P-Tyr) or an anti-LYN antibody. (**C**) Primary MCL cells (UPN3, UPN14) were treated with variable concentrations of PP2 (2 to 20 μM) or dasatinib (1 to 200nM) for 2 h. Phospho-Tyr397 LYN and LYN total were analyzed by western-blot. (**D**) Primary MCL cells were treated with various concentrations of PP2 (UPN3, *top panel*) or 10 μM of PP2 (UPN 1–3-7–8-9–14*, middle panel*) for 24 h and apoptosis was measured by flow cytometry after gating on CD19+ cells. All measurements were done in duplicate and the mean is provided. Results are also shown as median ± quartile (*box*) ± SE (*bars*) *bottom panel*). Differences between groups were determined using the paired Student *t* test. (**E**) Primary MCL cells were treated with dasatinib for 24 h with various concentrations *(top panel)* or with 100nM *(middle panel).* Apoptosis was measured as described above. Results are also shown as median ± quartile (*box*) ± SE (*bars*) *bottom panel*).

The BCR signal is initially transmitted by LYN kinase leading to activation of various signaling pathways including JNK. We therefore evaluated the activation status of LYN in MCL cells and its involvement in cell survival. Using an anti-phospho-SFK recognizing the catalytic site of several Src kinases among which the Tyr397 of LYN, we detected in 9 out of 10 UPN cases tested (UPN 1,3,5,7,8,9,10,13,14) such a specific signal to variable extents of constitutive phosphorylation forming a 53–56 kDa doublet (Figure 4A and Additional file 3: Figure S2). We confirmed that this doublet corresponded to phospho-LYN by an immunoprecipitation assay using an anti-LYN antibody (Figure 4B). Considering the constitutive activation of LYN in MCL cells, we next evaluated the impact of PP2, a synthetic pyrazolopyrimidine selective inhibitor of SFK, and dasatinib (BMS-354825), an oral multi kinase inhibitor which also inhibits the trans-autophosphorylation of the active Tyr397 residue of LYN [30]. Treatment of primary cells with PP2 or dasatinib led to a dose-dependent decrease of Tyr397 LYN phosphorylation and complete inhibition was achieved up to 10 μM and 100nM for PP2 and dasatinib respectively (Figure 4C). Inhibition of phospho-Tyr397 LYN by PP2 was associated with a significant and dose-dependent increase of apoptosis rate (from 49±4% to 67±3% apoptotic cells for untreated and 24 h treated (10 μM) cells respectively; p=0.006; n=6) (Figure 4D). Treatment with dasatinib for 24 h also led to a significant and dose-dependent increase of apoptosis (from 46±5% to 64±5% apoptotic cells for untreated and treated (100nM) cells, respectively; p=0.0001; n=7) (Figure 4E). Remarkably, dasatinib had little apoptosis effect on phospho-Tyr397 LYN-negative cells (UPN4) at a concentration up to 200nM (Figure 4E, *top panel*). Altogether, these results indicate that MCL cells display a constitutive phosphorylation of BCR-associated LYN and that treatment with dasatinib or PP2 suppressed LYN activation and increased spontaneous apoptosis.

### Inhibition of the BCR-induced LYN phosphorylation by PP2 or dasatinib is associated with a suppression of BCR-mediated cell survival

**Figure 5:**
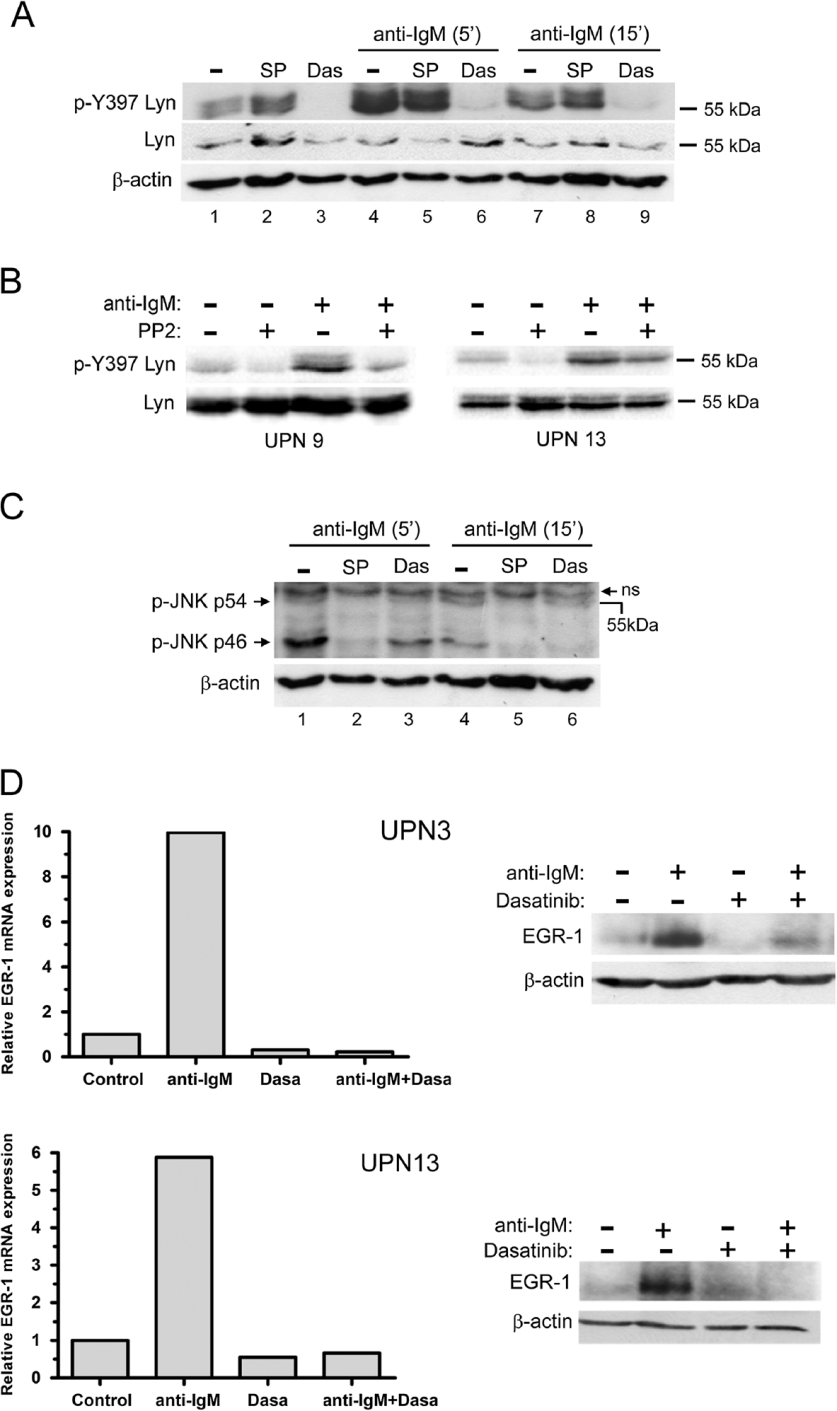
**PP2 and dasatinib inhibit BCR-induced LYN and JNK activation and EGR-1 upregulation.** (**A**) Patients’ cells (UPN9) were pretreated with dasatinib (100nM) or SP600125 (10 μM) for 1 h and stimulated for 5 min or 15 min with soluble anti-IgM (10 μg/ml). Phospho-Tyr397 LYN was detected using a pan phospho-src family antibody. (**B**) The same experiment was done with PP2 (10 μM) on UPN 9 and UPN 13 under the same conditions of BCR stimulation for 10 min. Lines 1 and 2 have to be compared to evidence the effect of PP2 on the constitutive level of phosphorylation for Lyn. Similarly lines 3 and 4 reflect this effect upon BCR stimulation. (**C**) BCR-induced phospho-JNK (p54 and p46) was analyzed under treatment with dasatinib (100nM) or SP600125 (10 μM) used herein as a positive control of phospho-JNK inhibition. (**D**) Impact of dasatinib on BCR-induced EGR-1 expression. MCL cells were pretreated with various concentrations of dasatinib as indicated and stimulated with immobilized anti-IgM. EGR-1 mRNA and protein were analyzed by qRT-PCR at 1 h of stimulation (*left panel*) and western-blot at 3 h of stimulation (*right panel*). Relative mRNA expression was calculated compared with unstimulated cells.

**Figure 6:**
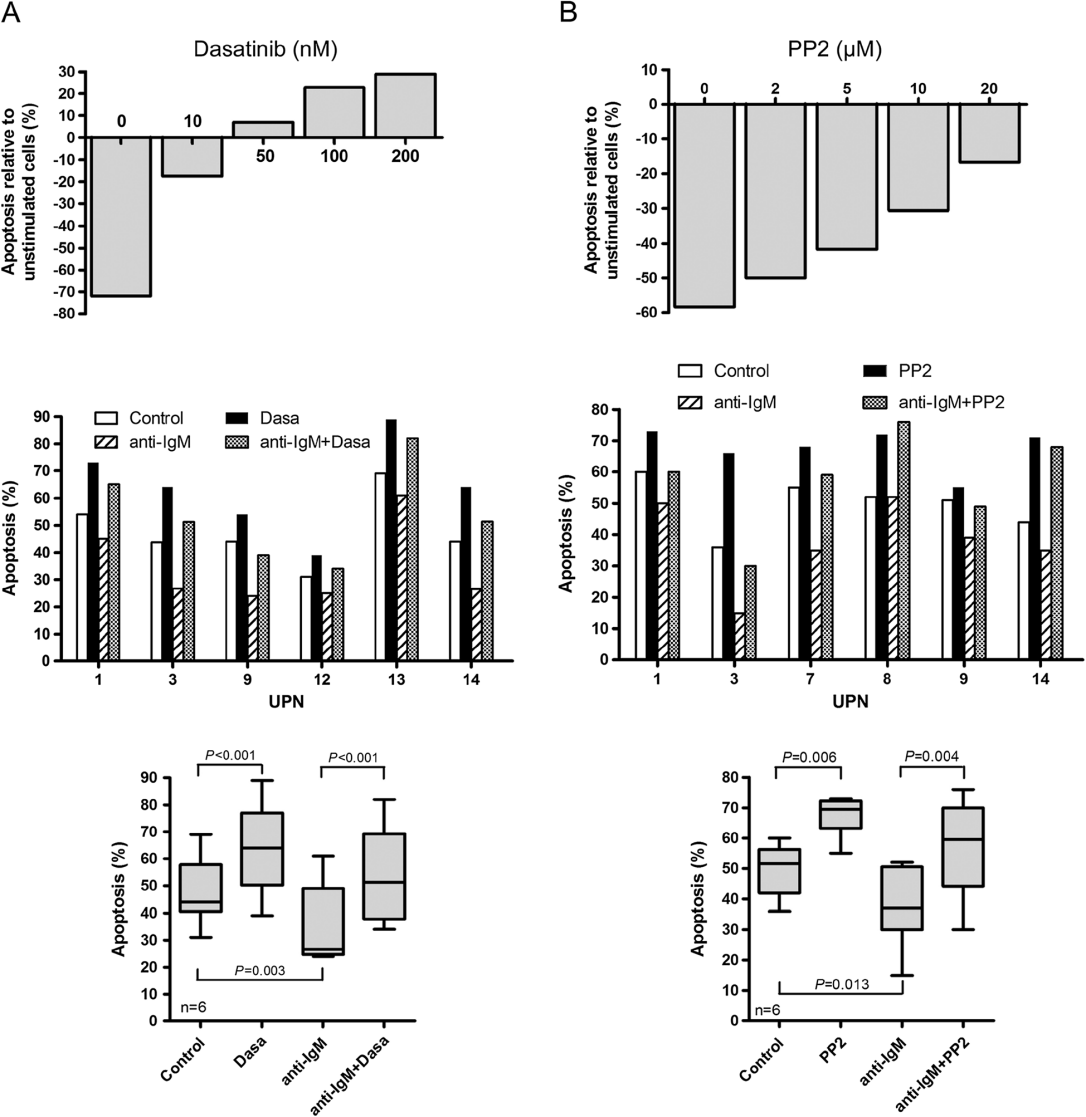
**PP2 and dasatinib suppress BCR-induced cell survival.** (**A**) Primary MCL cells (UPN3) were either left untreated or stimulated for 24 h with an anti-IgM antibody in the presence or in the absence of various concentrations of dasatinib (10 to 200nM) *(top panel)*. Apoptosis rates (Annexin V +/PI+ cells) were measured by flow cytometry after gating on CD19+ cells. For each BCR-stimulated condition (± dasatinib), the percentage of apoptotic cells was normalized to unstimulated cells and calculated as follows: [(% apoptosis BCR stimulated cells -% apoptosis BCR-unstimulated cells) / (% apoptosis BCR-unstimulated cells)] x100*. Middle panel:* Apoptosis rates from 6 MCL cases were measured from unstimulated or BCR-stimulated cells (24 h) either in absence or presence of 10 nM dasatinib. All measurements were done in duplicate and the mean is provided. Results are also shown as median ± quartile (*box*) ± SE (*bars*) (n=6) (*bottom panel*). Differences between groups were determined using the paired Student *t* test. (**B**) Primary cells were treated with PP2 according to the same protocol described in (**A**).

Since PP2 and dasatinib efficiently blocked activation of BCR-associated LYN in MCL cells, we next evaluated the impact of these compounds on JNK phosphorylation, EGR-1 expression and on cell survival upon BCR engagement. As shown in Figure 5A, a strong increase of phospho-Tyr397 LYN was observed in response to BCR ligation (lane 1 compared to lane 4) and treatment with dasatinib (100nM) completely blocked this effect while SP600125 that affect JNK did not. Similarly, PP2 decreased BCR-induced phospho-Tyr397 LYN in primary MCL cells (Figure 5B). Dasatinib also reduced BCR-induced phospho-JNK p46 (Figure 5C, lane 1 compared to lane 3 and lane 4 compared to lane 6), positioning JNK as a downstream target of LYN in response to BCR engagement. We next evaluated the impact of dasatinib on basal and BCR-induced level of EGR-1 as a target of JNK. As shown in Figure 5D (*left panels*), dasatinib (100nM) decreased basal expression of EGR1 mRNA and totally abrogated its upregulation in response to BCR ligation (UPN3, UPN13). Dasatinib also slightly decreased basal level of EGR1 protein and blocked its BCR-induced upregulation (Figure 5D, *right panels* and Additional file 4: Figure S3). Finally, we evaluated the impact of PP2 and dasatinib treatment on BCR-induced cell survival. Increasing concentrations of dasatinib abrogated the BCR-induced survival response in a dose-dependent manner and significantly suppressed this survival signal in all UPN cases tested (from 34 ±6% to 54±7% apoptotic cells for untreated and dasatinib-treated BCR-activated cells, respectively; n=6; p<0.001) (Figure 6A). Similarly, PP2 treatment also reduced or abolished BCR-induced cell survival (Figure 6B). Overall, these results highlight the importance of LYN, JNK and EGR1 as intermediates of BCR signaling in mediating survival signals in MCL cells and point out to the efficiency of dasatinib in suppressing cell survival signal emanating from the BCR.

## Discussion

In the present study, we showed that primary MCL cells displayed a constitutive and BCR-induced activation of LYN and that treatment with dasatinib or with a more specific inhibitor of LYN suppressed both BCR-induced JNK phosphorylation and EGR-1 upregulation and is associated with a decrease of cell survival.

Recent studies have shown the importance of tonic BCR signaling in survival of DLBCL cells [10] and CLL cells [11] but few studies focused on the role of BCR signaling in MCL cell survival [14,15]. We have previously shown in MCL cells that BCR engagement induced a cell survival signal through an IL6/IL10 autocrine dependent activation of STAT3 [14]. To further identify early genes involved in BCR-induced survival, we looked at the differential gene expression upon BCR stimulation. We evidenced that BCR engagement led to a rapid but transient induction of mRNA and protein levels of EGR-1. EGR-1 is a zinc finger transcription factor whose expression has been described as directly dependent on antigen receptor signaling [31,32]. EGR-1 is a downstream target of JNK and it regulates the expression of several genes like CD44, NF-kB1, thymidine kinase, cyclin D1 and platelet-derived growth factor that are important for cell survival and proliferation [23,33-35]. We thus evaluated the role of EGR-1 in MCL cell survival and showed that inhibition of JNK by SP600125 induced a decrease of constitutive and BCR-induced EGR-1 expression, associated with an increase of apoptosis and a suppression of BCR-induced survival. We confirmed the JNK-dependent upregulation of EGR-1 by blocking the activity of TAK1, the upstream activator of JNK, which was recently described to play an essential role in MCL survival [36]. Our results indicate that in MCL cells, EGR-1 is a downstream target of BCR signaling and its expression can be enhanced in response to antigen stimulation leading to cell survival. In addition to EGR-1, we observed that the BCR engagement also led to an increase of c-MYC in patients’ cells only. This differential response between cell lines and primary cells might reflect higher levels of c-MYC expression in cell lines as compared to patient’s cells [37]. Cell lines might therefore become unresponsive to further stimulation through the BCR. The delayed kinetic induction of c-Myc as compared to EGR-1 in patient’s cells might argue for a latter induction of c-Myc. Whether this induction is related to expression of EGR-1 as proposed in CLL [38] and BKS2 cells activated by CpG ODN [39] remains to be determined. Nevertheless, our results suggest that EGR-1 and c-MYC upregulations could play an essential role in BCR-induced survival of MCL cells*.*

The importance of BCR signaling in MCL was recently investigated using a high-throughput phospho-proteomic technique which identified more than 300 tyrosine-phosphorylated proteins [15]. The most abundant peptides were part of proteins constituting the BCR-associated signalosome. Among them, the kinases LYN and SYK were found to be constitutively strongly phosphorylated, thus reflecting an active BCR signaling even in absence of antigen stimulation. The importance of the BCR signaling in MCL was also suggested through the activation of SYK probably due to a constitutively activated signalosome made of LYN and CSK-binding protein/phosphoprotein associated with glycosphingolipid-enriched microdomains (Cbp/PAG) membrane adaptor [6,40]. In the present study, we showed in a subset of primary MCL cells that LYN was in a constitutively active form as revealed by phosphorylation of the active Tyr397 LYN residue. LYN is believed to be a key component of cell membrane lipid rafts. Moreover, a subset of transmembrane proteins with aberrant expression was identified in MCL plasma membranes [41]. In particular, Cbp/PAG that participates to the negative regulation of LYN in resting B cells through CSK recruitment was underexpressed in MCL primary cells compared to normal B cells. This low expression of Cbp/PAG could thus contribute to the constitutive activation of LYN in MCL cells.

Dasatinib, a dual BCR/ABL and SFK inhibitor, has demonstrated its efficacy in inhibiting cell proliferation of lymphoma B cells exhibiting a constitutive activation of Src kinase. Yang *et al*. demonstrated that inhibition of the Src-SYK-PLCg2 pathway by dasatinib induced G1 arrest in DLBCL [42]. In the present study, we evidenced a constitutive and BCR-induced phosphorylation of LYN, thus justifying the rationale to evaluate the impact of dasatinib in MCL cell survival. We showed that dasatinib, which targeted the ATP binding pocket of LYN [30], inhibited phosphorylation of Tyr397 LYN probably by blocking its trans-autophosphorylation. .We also showed for the first time that dasatinib induced apoptosis of primary MCL cells and suppressed BCR-induced survival after antigen-triggering at nanomolar range. Of interest, the concentration levels of dasatinib (100nM) required to induce *in vitro* MCL cell apoptosis are in agreement with clinically achievable doses [43]. A phase II study of dasatinib in relapsed or refractory CLL showed partial responses in 3 of 15 patients and among the remaining 12 patients, five patients had nodal responses. The investigators thus concluded that dasatinib as a single agent had activity in relapsed and refractory CLL [44]. A phase I/II study of dasatinib is currently conducted by recruiting patients in relapsed or refractory non-hodgkin’s lymphoma (NHL) including mantle cell lymphoma (NCT00550615).

## Conclusion

In conclusion, this study performed on primary MCL lymphocytes evidenced a dysregulation of early BCR signaling characterized by a constitutive LYN phosphorylation which can be enhanced in response to BCR engagement. Furthermore, targeting proximal BCR-associated kinases efficiently induced apoptosis of MCL cells. Thus, inhibition of LYN kinase and downstream JNK/EGR-1 pathway could be a new therapeutic strategy in MCL to overcome pro-survival signal emanating from the BCR.

## Methods

### MCL samples and cell lines

Peripheral blood mononuclear cells were obtained from 14 MCL leukemic patients by Ficoll-Hypaque density gradient (Stem Cell Technologies, Grenoble, France). Lymphocytosis was greater than 8.0×10^9^/L and 10 out of 14 samples contained at least 80% of B lymphocytes (Additional file 5: Table S1). All B lymphocytes are monoclonal tumor B cells as evidenced through flow cytometry phenotyping of the surface immunoglobulin light chain (monotypic kappa or lambda). Seven cases (7/14) showed mutated IGHV and none of them displayed mutation in ITAM sequences of CD79B. The diagnosis of MCL was ascertained by immunophenotyping, cytogenetic and FISH analysis of t(11;14) and overexpression of cyclin D1 was detected by competitive RT-PCR according to the World Health Organization classification. All patients were homogeneously treated in a prospective trial of the GOELAMS group “Lymphome du manteau 2006 SA” [45]. All patients were provided written informed consent, validated by the Ethics Committee from the GOELAMS group, in accordance with the Declaration of Helsinki. Patients usually received treatment very quickly after sampling, making it difficulties to repeat all experiments several times on the same sample. Jeko-1, and Granta-519 cell lines were purchased from the German Collection of Microorganisms and Cell Cultures (DSMZ, Braunshwieig, Germany) and the HBL-2 cell line was a generous gift from Dr B. Sola (Caen, France) [46].

Patients’ cells were either used freshly isolated or cryopreserved in liquid nitrogen in the presence of 10% dimethyl sulfoxide and 20% heat-inactivated FCS. MCL leukemic cells (3 × 10^6^ cells/ml) were cultured in complete RPMI 1640 medium supplemented with 10% heat-inactivated foetal calf serum. Jeko-1, HBL-2 and Granta-519 cell lines were maintained in culture in the same media. For BCR stimulation, plates were pre-coated with rabbit anti-human IgM antibody (10 μg/mL; Jackson ImmunoResearch, Baltimore, MD) as previously described [14] or the anti-IgM antibody was added to the culture medium at the same concentration for short stimulation time.

### Antibodies and reagents

Antibodies to EGR-1 (mAb 44D5), c-MYC (mAb 9B11), phospho-Src family (mAb 100 F9) also reactive with phospho-Tyr397 LYN (catalytic site) and phospho-JNK (Thr183/Tyr185) were from Cell Signaling (Beverley, MA). Monoclonal mouse antibody (H-6) and polyclonal rabbit antibody (44) to LYN were from Santa-Cruz (Santa Cruz, CA). Anti-phosphotyrosine monoclonal antibody (clone 4 G10*)* was from Millipore (Billerica, MA). Dasatinib (Clini Sciences, Montrouge, France) was used at 100nM, unless otherwise stated. JNK inhibitor SP600125 and PP2 (4-Amino-3-(4-chlorophenyl)-1-(t-butyl)-1H-pyrazolo[3,4-d]pyrimidine) was from Sigma (Saint Quentin Fallavier, France) and (5Z)-7-Oxozeaenol (TAK1 inhibitor) was from Tocris Bioscience (Bristol, UK).

### RT^2^ profiler PCR arrays

Tumor B-lymphocytes from MCL patients were purified by the RosetteSep® Human B Cell Enrichment Cocktail (Stemcell technologies, Grenoble, France). Cells were cultured for 3 hours upon BCR stimulation or left untreated. Total RNA were extracted and analyzed with “p53 signaling pathway” array (SA Biosciences PAHS-027) according to the manufacturer’s instructions (SA Biosciences, Frederick, MD) with an Applied Biosystems 7500 Fast Real-Time PCR Systems. Each gene expression was normalized to the mean Ct values from the four housekeeping genes available in the PCR array (β2-microglobulin, β-actin ribosomal protein L13A, hypoxanthine phosphorybosyl transferase-1 and β-actin), then normalized to unstimulated control cells to determine the fold-change. Relative fold change of expression was calculated by the ΔΔCt method and the values are expressed as 2^-ΔΔCt^. All points were done in duplicate.

### Apoptosis assay

Cell apoptosis was evaluated using flow cytometry (FACSCantoTM II Becton Dickinson) on leukemic MCL PBMC after gating on CD19+ cells using Annexin V-FITC and propidium iodide staining (BD Biosciences, San Jose, CA). Percentage of apoptotic cells corresponded to% of annexin V-positive, including PI-negative and PI-positive cells. All measurements were done in duplicate and the mean is indicated.

### Quantification of EGR-1 and c-MYC mRNA by qRT-PCR

RNA from unstimulated or anti-IgM stimulated cells were extracted using RNeasy Mini kit (QIAGEN) and EGR-1 and c-MYC expressions were analyzed by qRT-PCR using SYBR Green reagents. Results were normalized to the mean Ct values from cyclophilin A housekeeping gene then normalized to unstimulated control cells to determine the fold change. Relative fold change of expression was calculated by the ΔΔCt method and the values are expressed as 2 ^-ΔΔCt^. All points were done in duplicate. The primers used for amplification (Sigma-Aldrich) were as follows:

EGR-1 forward primer (5^′^CGAGCAGCCCTACGAGCACCTGAC3^′^),

EGR-1 reverse primer (5^′^TGCGCAGCTCAGGGGTGGGCTCTG3^′^),

c-MYC forward primer (5^′^GCTGCTTAGACGCTGGATTTTT3^′^),

c-MYC reverse primer (5^′^ACCGAGTCGTAGTCGAGGTCAT3^′^),

cyclophilin A forward primer (5^′^GCACTGGAGAGAAAGGATTTGG3^′^) and

cyclophilin A reverse primer (5^′^AGTGCCATTATGGCGTGTGA3^′^).

### Western blotting and immunoprecipitation

Total protein extracts from 3×10^6^ MCL cells (PBMC) were separated on 10% polyacrylamide denaturing gel, transferred to a nitrocellulose membrane and incubated overnight with the appropriate antibody followed by a secondary horseradish peroxidase-conjugated antibody (Bio-Rad). Detection was performed using ECL (Amersham Biosciences, Buckinghamshire, UK) and autoradiography (Biomax, Kodak, Rochester, USA). For LYN immunoprecipitation, HBL-2 cells were lysed in 1% Nonidet P-40 (NP-40) lysis buffer for 30 minutes on ice. Insoluble material was removed by centrifugation at 27 000 g for 10 minutes at 4°C and soluble proteins were immunoprecipitated with a rabbit anti-LYN (H-6) antibody for 2 hours at 4°C. Immunocomplexes were solubilized in SDS sample buffer, analyzed on SDS-PAGE, transferred and subjected to immunoblotting as described above using either a mouse anti-phosphotyrosine antibody (clone 4 G10*)* or a mouse anti-LYN (44) antibody.

### siRNA assay

Three million cells were resuspended in 100 μL of Human B Cell Lymphoma Nucleofector® Kit (Amaxa) containing either 1 μM of EGR-1 siRNA (On-Target plus SMART pool Human EGR-1, Dharmacon) or 1 μM of control siRNA (Ctrl1: On-Target plus non-targeting pool; Ctrl2: On-target plus non-targeting siRNA#1; Dharmacon). Cells were transfected in a Nucleofector II device (Lonza) by using U-015 program, transferred to culture plates and western blot and apoptosis assays were performed as described above.

### Statistical analyses

Differences between groups were determined using the Student’s *t* test. Statistical analyses were performed using GraphPad Prism software (San Diego, CA). *P* values below 0.05 were considered statistically significant.

## Abbreviations

MCL: Mantle cell lymphoma; SFK: Src family kinase; BCR: B-cell receptor; JNK: c-JUN NH2-terminal kinase; EGR-1: Early growth response gene-1; IGHV: Immunoglobulin heavy chain variable-region; MAPK: Mitogen-activated protein kinases; ERK: Extracellular signal-regulated kinase; CLL: Chronic lymphocytic leukemia; DLBCL: Diffuse large B cell lymphoma; PBMC: Peripheral blood mononuclear cells; TAK1: Transforming growth factor-β activated kinase-1; ITAM: Immunoreceptor tyrosine-based activation motif; SYK: Spleen tyrosine kinase; MYC: Myelocytomatosis; SH2: Src-homology 2; PLC: Phospholipase C; PKC: Protein kinase C; IL: Interleukin; STAT3: Signal transducer and activator of transcription 3

## Competing interests

The authors declare that they have no conflict of interest.

## Authors’ contribution

MB, JT, CR performed research. RG, AM provided reagents and members of the GOELAMS group. NVB and FC contributed reagents and helped to design the study and to write the manuscript. DL designed research, analyzed results, made the figures and wrote the manuscript. FBM designed research, performed experiments, analyzed results, made the figures and wrote the manuscript. All authors read and approved the final manuscript.

## Authors’ information

This work was supported by the GOELAMS group (Groupe Ouest-Est des Leucémies Aiguës et Maladies du Sang.) and by the Institut National du Cancer (INCA) for the tumor biobank. M.A.B. was supported by the Société Française d’Hématologie (SFH).

FBM and DL are co-senior authors.

## Supplementary Material

Additional file 1: Table S2 Differentially expressed genes in BCR-stimulated MCL cells (3 h) compared with unstimulated cells. Click here for file

Additional file 2: Figure S1 Inhibition of TAK1 protein by (5Z)-7-Oxozeaenol suppressed BCR-induced EGR1 expression. HBL2 cells were pretreated with (5Z)-7-Oxozeaenol (5Z-7-oxo) (0.3 and 0.5 μM) for 1 h and then stimulated with immobilized anti-IgM (10 μg/ml). Total protein extracts were analysed by western-blot for EGR1 expression. Click here for file

Additional file 3: Figure S2 Constitutive phosphorylation of LYN in primary MCL cells. Total protein from UPN1, UPN5, UPN13 and UPN14 were extracted and analysed by western blot. Phospho-Tyr397 LYN was detected using a pan phospho-src family antibody. The blots were stripped and re-probed for total LYN. Click here for file

Additional file 4: Figure S3 Dasatinib treatment suppresses BCR-induced upregulation of EGR-1 protein. HBL-2 cells were pretreated (1 h) with various concentrations of Dasatinib (1nM-200nM) and stimulated with immobilized anti-IgM for 1 h (anti-IgM) or left unstimulated (−). EGR1 protein level was then analysed by western blot. Click here for file

Additional file 5: Table S1 Characteristics of the 14 MCL cases (UPN). Click here for file
