# Supplementary material for: Targeting early B-cell receptor signaling induces apoptosis in leukemic mantle cell lymphoma
Source: Exp Hematol Oncol. 2013 Feb 19;2:4. doi: 10.1186/2162-3619-2-4 (PMC3585857; doi:10.1186/2162-3619-2-4)
Supplement: Additional file 1: Table S2 — Differentially expressed genes in BCR-stimulated MCL cells (3 h) compared with unstimulated cells. [file 2162-3619-2-4-S1.doc]

**Supplementary Table S2.** Differentially expressed genes in BCR-stimulated MCL cells (3h) compared with unstimulated cells.

| Gene name | Description | Average fold change^§^ |
| --- | --- | --- |
| APAF1 | Apoptotic peptidase activating factor 1 | -3.4 |
| CCNG2 | Cyclin G2 | -2.9 |
| ATM | Ataxia telangiectasia mutated | -1.7 |
| BTG2 | BTG family, member 2 | -1.6 |
| CDK4 | Cyclin-dependent kinase 4 | 2.0 |
| MCL1 | Myeloid cell leukemia sequence 1 (BCL2-related) | 2.2 |
| IFNB1 | Interferon, beta 1 | 2.3 |
| SESN2 | Sestrin 1 | 2.3 |
| NFKB | Nuclear factor of kappa light polypeptide gene enhancer in B cells 1 | 2.3 |
| BCL2A1 | BCL2-related protein A1 | 2.8 |
| IL6 | Interleukin 6 | 3.0 |
| TNF | Tumor necrosis factor | 4.6 |
| HK2 | Hexokinase 2 | 5.4 |
| EGR1 | Early growth response 1 | 17.6 |
| MYC | V-myc myelocytomatosis viral oncogene homolog | 23.6 |

^§^qPCR arrays were performed on purified CD19+/CD5+ tumor cells and fold change represents the average value from 4 MCL samples (UPN 5, 10, 13 and 14)
