# Supplementary material for: Targeting early B-cell receptor signaling induces apoptosis in leukemic mantle cell lymphoma
Source: Exp Hematol Oncol. 2013 Feb 19;2:4. doi: 10.1186/2162-3619-2-4 (PMC3585857; doi:10.1186/2162-3619-2-4)
Supplement: Additional file 2: Figure S1 — Inhibition of TAK1 protein by (5Z)-7-Oxozeaenol suppressed BCR-induced EGR1 expression. HBL2 cells were pretreated with (5Z)-7-Oxozeaenol (5Z-7-oxo) (0.3 and 0.5 μM) for 1 h and then stimulated with immobilized anti-IgM (10 μg/ml). Total protein extracts were analysed by western-blot for EGR1 expression. [file 2162-3619-2-4-S2.doc]

**Supplementary figure S1**


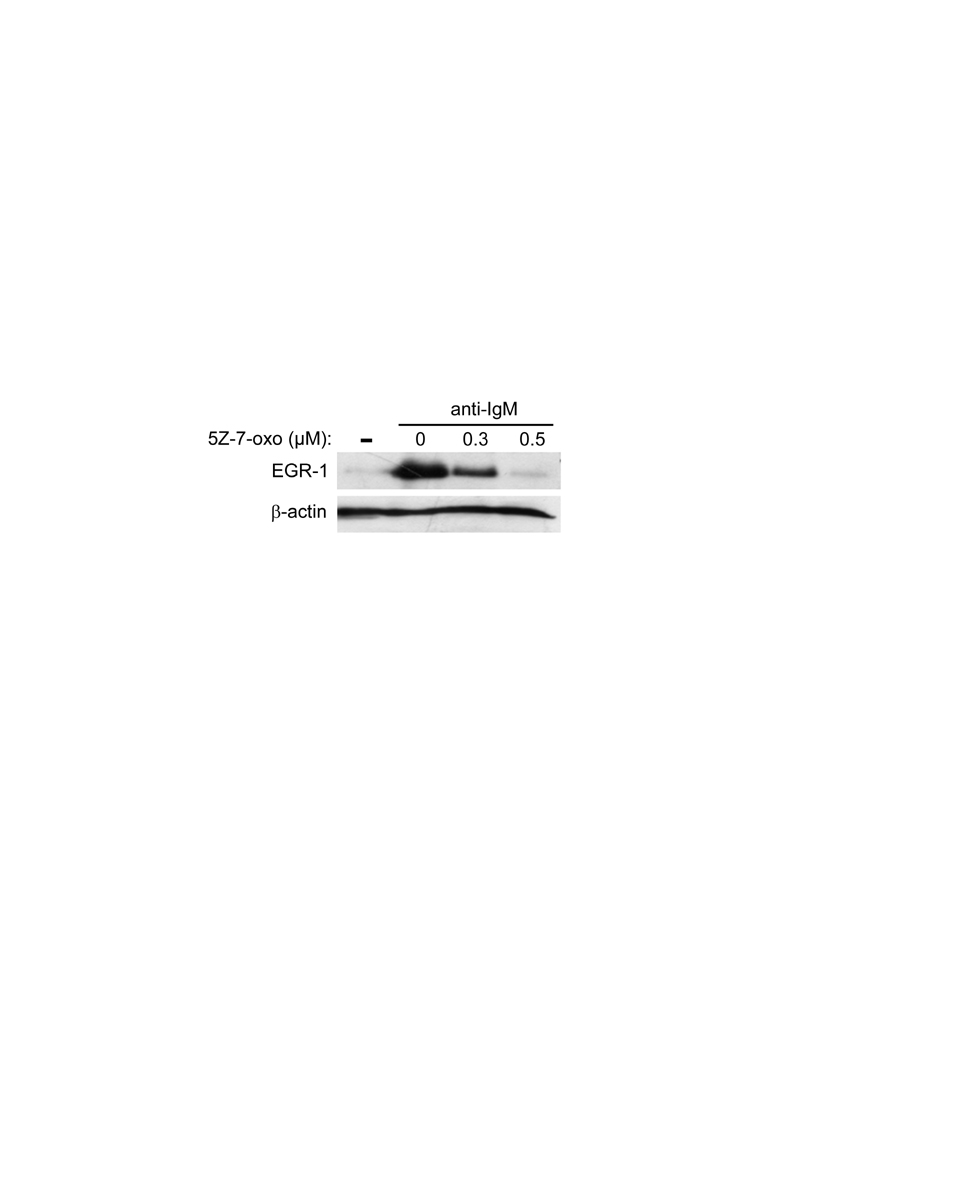


**Inhibition of TAK1 protein by (5Z)-7-Oxozeaenol** **suppressed BCR-induced EGR1 expression.** HBL2 cells were pretreated with (5Z)-7-Oxozeaenol (5Z-7-oxo) (0.3 and 0.5 µM) for 1h and then stimulated with immobilized anti-IgM (10µg/ml). Total protein extracts were analysed by western-blot for EGR1 expression.
