# Supplementary material for: Targeting early B-cell receptor signaling induces apoptosis in leukemic mantle cell lymphoma
Source: Exp Hematol Oncol. 2013 Feb 19;2:4. doi: 10.1186/2162-3619-2-4 (PMC3585857; doi:10.1186/2162-3619-2-4)
Supplement: Additional file 3: Figure S2 — Constitutive phosphorylation of LYN in primary MCL cells. Total protein from UPN1, UPN5, UPN13 and UPN14 were extracted and analysed by western blot. Phospho-Tyr397 LYN was detected using a pan phospho-src family antibody. The blots were stripped and re-probed for total LYN. [file 2162-3619-2-4-S3.doc]

**Supplementary figure S2**


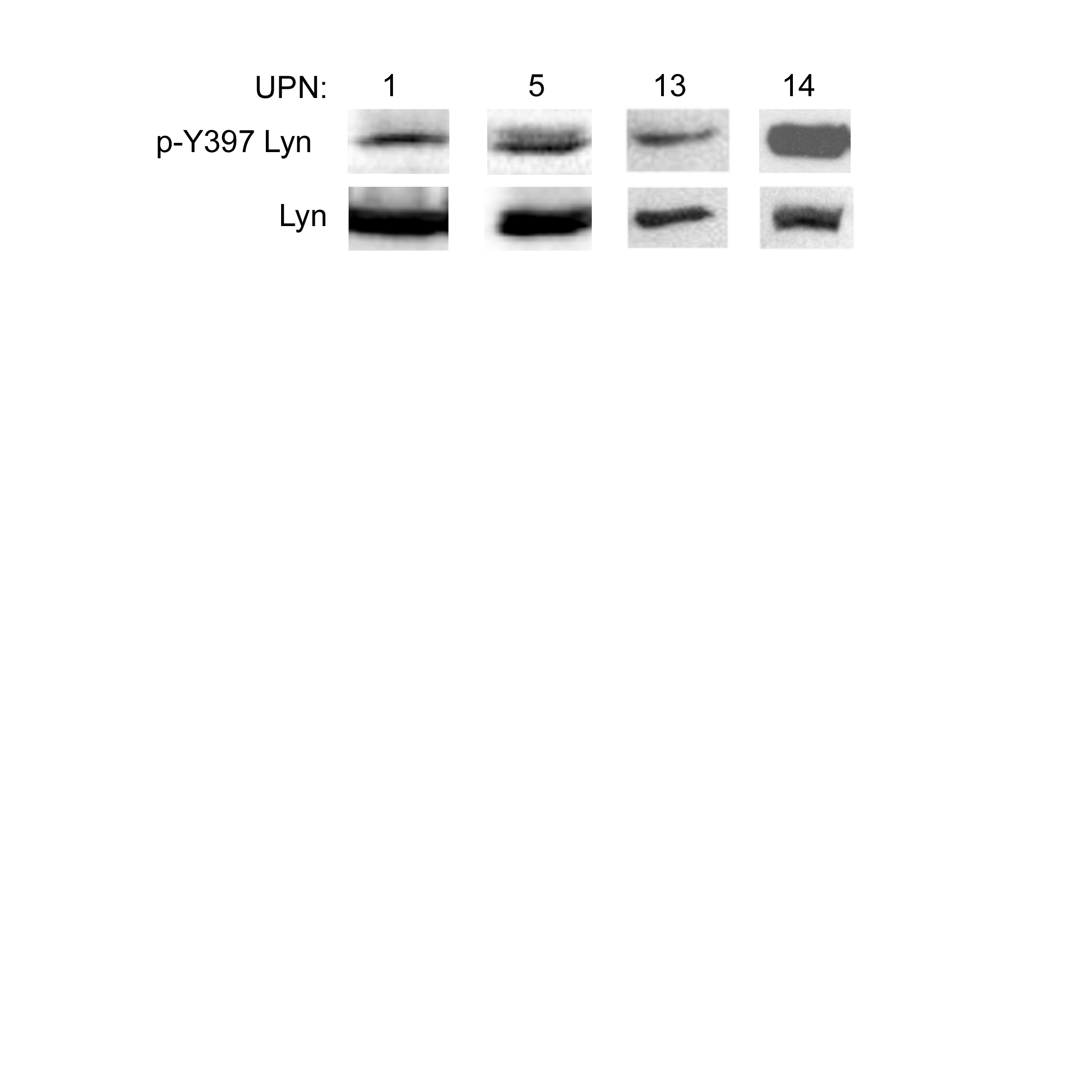


**Constitutive phosphorylation of LYN in primary MCL cells.** Total protein from UPN1, UPN5, UPN13 and UPN14 were extracted and analysed by western blot. Phospho-Tyr397 LYN was detected using a pan phospho-src family antibody. The blots were stripped and re-probed for total LYN.
