# Supplementary material for: Targeting early B-cell receptor signaling induces apoptosis in leukemic mantle cell lymphoma
Source: Exp Hematol Oncol. 2013 Feb 19;2:4. doi: 10.1186/2162-3619-2-4 (PMC3585857; doi:10.1186/2162-3619-2-4)
Supplement: Additional file 4: Figure S3 — Dasatinib treatment suppresses BCR-induced upregulation of EGR-1 protein. HBL-2 cells were pretreated (1 h) with various concentrations of Dasatinib (1nM-200nM) and stimulated with immobilized anti-IgM for 1 h (anti-IgM) or left unstimulated (−). EGR1 protein level was then analysed by western blot. [file 2162-3619-2-4-S4.doc]

**Supplementary Figure S3**


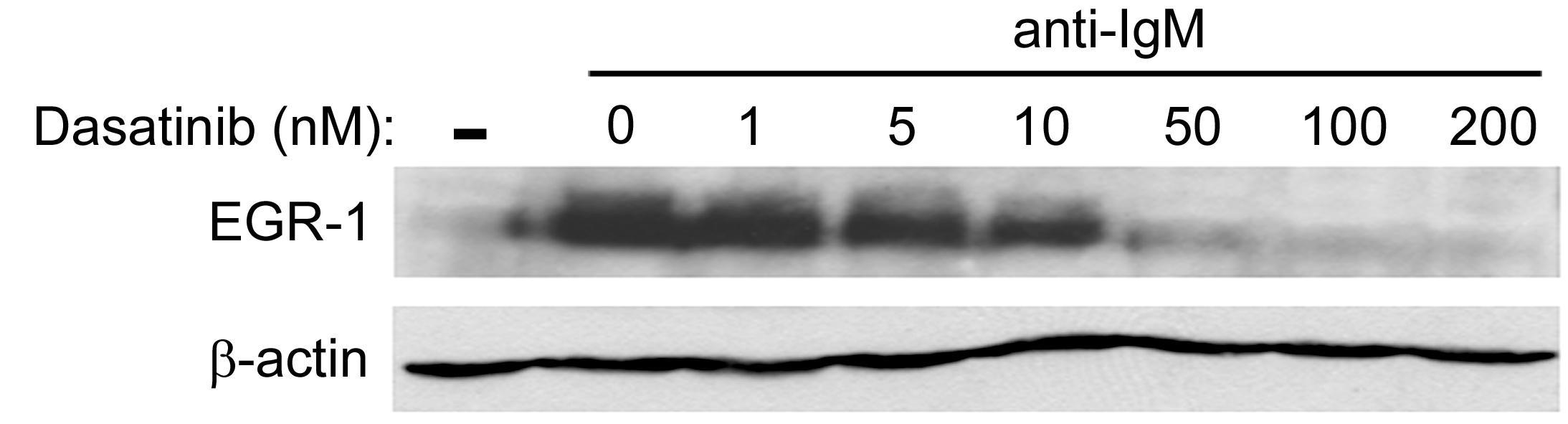


**Dasatinib treatment suppresses BCR-induced upregulation of EGR-1 protein.** HBL-2 cells were pretreated (1h) with various concentrations of Dasatinib (1nM-200nM) and stimulated with immobilized anti-IgM for 1h (anti-IgM) or left unstimulated (-). EGR1 protein level was then analysed by western blot.
