# Supplementary material for: Targeting early B-cell receptor signaling induces apoptosis in leukemic mantle cell lymphoma
Source: Exp Hematol Oncol. 2013 Feb 19;2:4. doi: 10.1186/2162-3619-2-4 (PMC3585857; doi:10.1186/2162-3619-2-4)
Supplement: Additional file 5: Table S1 — Characteristics of the 14 MCL cases (UPN). [file 2162-3619-2-4-S5.doc]

Supplementary Table S1. Characteristics of the 14 MCL cases (UPN).

| UPN | Sex | Age | IGHV mutational status† | Lymphocytosis giga/L | % tumor cells^§^ |
| --- | --- | --- | --- | --- | --- |
| 1 | F | 85 | M | 189,3 | 90 |
| 2 | M | 71 | M | 158,9 | 96 |
| 3 | M | 56 | M | 50,22 | 90 |
| 4 | M | 59 | M | 15,04 | 77 |
| 5 | M | 68 | UM | 42,91 | 86 |
| 6 | M | 71 | UM | 342,7 | 90 |
| 7 | M | 86 | UM | 119 | 90 |
| 8 | M | 75 | UM | 50,51 | 90 |
| 9 | M | 69 | M | 16,58 | 70 |
| 10 | F | 63 | M | 10,43 | 60 |
| 11 | M | 58 | UM | 8,6 | 60 |
| 12 | M | 59 | M | 29,7 | 80 |
| 13 | M | 57 | UM | 46,8 | 90 |
| 14 | M | 61 | UM | 25,01 | 80 |

† Unmutated (UM): IGHV homology ≥ 98%; Mutated (M): IGVH homology < 98%

§ Percentage of CD19+/CD5+ tumor cells in PBMC sample.
